# The Effect of Loop Diuretics on 28-Day Mortality in Patients With Acute Respiratory Distress Syndrome

**DOI:** 10.3389/fmed.2021.740675

**Published:** 2021-09-21

**Authors:** Rui Zhang, Hui Chen, Zhiwei Gao, Meihao Liang, Haibo Qiu, Yi Yang, Ling Liu

**Affiliations:** ^1^Jiangsu Provincial Key Laboratory of Critical Care Medicine, Department of Critical Care Medicine, School of Medicine, Zhongda Hospital, Southeast University, Nanjing, China; ^2^Department of Critical Care Medicine, The First Affiliated Hospital of Soochow University, Suzhou, China; ^3^Department of Critical Care Medicine, Huai'an First People's Hospital, Nanjing Medical University, Huai'an, China

**Keywords:** acute respiratory distress syndrome, diuretics, mortality, marginal structural cox model, subtype

## Abstract

**Background:** Diuretics have been widely used in critically ill patients while it remains uncertain whether they can reduce mortality in patients with acute respiratory distress syndrome (ARDS). This study aimed to investigate the associations between diuretics and 28-day mortality in patients with ARDS.

**Methods:** This is a secondary analysis of the ARDS Network Fluid and Catheter Treatment Trial (FACTT) of National Heart, Lung, and Blood Institute. Those patients who did not receive renal replacement therapy within the first 48 h after enrollment in the FACTT were included in the analysis. A marginal structural Cox model (MSCM) was used to investigate the associations between diuretics and 28-day mortality after correction of both the baseline and time-varying variables. The latent class analysis (LCA) and subgroup analysis were performed to identify the kind of patients that could be benefited from diuretics.

**Results:** A total of 932 patients were enrolled, i.e., 558 patients in the diuretics group and 374 patients in the no diuretics group within the first 48 h. The 28-day mortality was lower in the diuretics group (15.1 vs. 28.1%, *p* < 0.001). In MSCM, diuretics use was related to the improved 28-day mortality (*HR* 0.78; 95% *CI* 0.62–0.99; *p* = 0.04). LCA identified three subtypes, and diuretics were associated with reduced mortality in subtype 3, which was characterized by worse renal function and higher central venous pressure (CVP). A subgroup analysis indicated survival advantage among the female patients, sepsis induced ARDS, and those with the ratio of partial pressure of oxygen to the fractional concentration of inspired oxygen (PaO_2_/FiO_2_) ≤ 150 mmHg, and mean arterial pressure (MAP) ≥ 65 mmHg.

**Conclusion:** Loop diuretics were associated with the reduced 28-day mortality in the patients with ARDS, after controlling for time-varying confounders. Randomized trials are required to verify the association.

## Introduction

Acute respiratory distress syndrome (ARDS) that results from various insults is associated with a high hospital mortality rate of 40% (1). The hallmark alteration in ARDS is increased endothelial and epithelial permeability, leading to the increased extravascular lung water (EVLW) (2), which is associated with lung injury and mortality (3). Diuretics are frequently administered to critically ill patients to alleviate pulmonary edema and may reduce lung injury (4).

Several studies have involved diuretics as part of therapeutic intervention for ARDS, but whether they could reduce mortality has not been conclusively determined. Diuretics have been associated with reduced positive fluid balance, improved lung function, and shorter mechanical ventilation duration, but no significant improvement in the mortality rate has been demonstrated (5, 6). One retrospective study suggested that the use of diuretics for 48–72 h after meeting the ARDS criteria may reduce mortality (7). In that study, the influence of diuretics use beyond the specified 24 h was not analyzed, and nor were therapeutic changes related to diuretics, rendering the result less explicable.

There are several theoretical reasons for the controversial results reported to date. ARDS is of extreme heterogeneity, and patients with diverse phenotypes respond differently to the selected treatment (8–10). In addition, the use of diuretics is a time-dependent variable that is affected by factors, such as oxygenation and mean arterial pressure, but these confounders were seldom corrected in studies, leading to bias. In a previous study, after adjusting for the baseline variables only, diuretics were associated with lower 28-day mortality in critically ill patients. When time-varying confounders were corrected *via* marginal structural Cox modeling (MSCM), however, there was no significant association (11). The study highlighted the necessity to consider time-dependent variables when investigating the effect of diuretics on patient outcomes.

Diuretics are widely used in critically ill patients, despite controversy with respect to whether they reduce mortality. The present study aimed to investigate the effects of loop diuretics on 28-day mortality in the patients with ARDS, and used a marginal structural model to adjust time-varying covariates. We hypothesized that diuretics would improve 28-day mortality in patients with ARDS. A latent class analysis (LCA) was used to derive phenotypes, and subgroup analysis was conducted to determine the phenotypes that may benefit from diuretics.

## Methods

### Study Design and Population

The study was a secondary analysis of the ARDS Network Fluid and Catheter Treatment Trial (FACTT) of the National Heart, Lung, and Blood Institute. The details of the trial have been published previously (5, 12). In the original study, the patients with ARDS who received mechanical ventilation were included. The patients with ARDS for more than 48 h, chronic diseases that impair survival and weaning were excluded from the study. We further excluded patients receiving renal replacement therapy routinely or within the first 48 h after enrollment, to whom diuretics were not likely to be prescribed.

Fluid management strategies were conducted for 7 days from randomization, or until weaning, whichever occurred first. Furosemide or other diuretics were administered to the patients with elevated central venous pressure (CVP) or pulmonary arterial wedge pressure when hemodynamics was stable. Patients were divided into two groups according to whether they received diuretics within the first 48 h. The primary outcome was the 28-day mortality. All data were obtained and approved by Biologic Specimen and Data Repository Information Coordinating Center (BioLINCC, https://biolincc.nhlbi.nih.gov). The present study was approved by the Research Ethics Commission of Zhongda Hospital, School of Medicine, Southeast University (Nanjing, China). The Strengthening the Reporting of Observational Studies in the Epidemiology (STROBE) recommendations were followed in this study.

### Data Collection

The data extracted included demographic data, laboratory tests, Acute Physiology and Chronic Health Evaluation III score (APACHE III), and prescriptions of vasopressor and diuretics. Sequential Organ Failure Assessment (SOFA) score, Charlson Comorbidity Index (13), and Murray lung injury score (14) were calculated. The number of missing or censoring values is presented in Supplementary Table 1. Variables with a missing ratio of more than 25% were not included in the final analysis. Outliers were censored and missing values were replaced by multiple imputations.

### Statistical Analysis

The continuous variables were presented as mean (SD) or median [interquartile ranges (IQR) and were compared with Student's *t*-test or the Mann–Whitney test. Categorical variables were compared via the chi-square test or Fisher's exact test. Standardized mean differences (SMDs) and *p*-values were calculated to evaluate the differences between the two groups.

Latent class analysis was employed to derive phenotypes. Variables were selected based on the previous research and potential association with outcomes (9, 15), such as demographic parameters (gender, age, and BMI), comorbidities (diabetes, hypertension, and heart failure), disease severity (APACHE III), vital signs (heart rate, temperature, and respiratory rate), hemodynamic parameters (MAP, CVP, vasopressor use, and fluid balance), respiratory variables (tidal volume and plateau pressure), hematology (platelet and hemoglobin), and the renal function indicator creatinine. Mplus (version 8.3) software was used to fit models with latent classes. The optimal number of classes was determined by a combination of Bayesian information criterion (BIC), entropy, and the Vuong-Lo-Mendell-Rubin (VLMR) test (16).

Daily fluid balance, MAP, need for vasopressors, and the ratio of partial pressure of oxygen to the fractional concentration of inspired oxygen (PaO_2_/FiO_2_), which would influence the decision of diuretics treatment and potentially correlate with the outcomes, were defined as time-dependent variables. The marginal structural model uses inverse probability of treatment-weighting (IPTW) estimator to create a pseudo-population, enabling the correction of time-fixed baselines and time-varying confounders (17, 18). MSCM was used to evaluate the effect of diuretics on 28-day mortality. Several specified subgroup analyses were performed. In this study, we used RStudio (version 1.3.1073, RStudio Inc., MA, USA) software to perform the statistical analyses. The variable *p* < 0.05 was deemed to indicate the statistical significance.

## Results

### Demographic and Clinical Characteristics

A total of 932 patients were included in the analysis, of which 558 (59.9%) received diuretics within the first 48 h since enrollment. The demographic and clinical characteristics of patients in the two groups are shown in Table 1. In general, patients in the diuretics group had less severe disease, higher mean arterial pressure, and a lower proportion of vasoactive agents use than those in the non-diuretics group. All-cause 28-day mortality was significantly lower in the diuretics group [84 (15.1%) vs. 105 (28.1%), *p* < 0.001], and a survival advantage was still evident at day 90. The detailed comparisons between the two groups are presented in Supplementary Table 2.

**Table 1:** Demographic and clinical characteristics of patients in two groups.

	**No diuretics**	**Diuretics**	***P***	**SMD**
*N*	374	558		
Age, years	50.20 (17.20)	49.58 (15.09)	0.561	0.038
Female (*n*, %)	172 (46.0)	261 (46.8)	0.866	0.016
Body mass index	28.93 (7.51)	28.77 (6.54)	0.801	0.023
APACHE III	97.81 (30.41)	88.20 (28.90)	<0.001	0.324
SOFA	8.57 (2.92)	7.35 (2.38)	<0.001	0.455
Primary lung injury (*n*, %)			<0.001	0.354
Sepsis	109 (29.1)	97 (17.4)		
Trauma	28 (7.5)	45 (8.1)		
Aspiration	52 (13.9)	94 (16.8)		
Pneumonia	171 (45.7)	267 (47.8)		
Other	14 (3.7)	55 (9.9)		
**Comorbidity (** ***n*** **, %)**				
Immune suppression	27 (7.2)	44 (7.9)	0.803	0.025
Diabetes	62 (16.6)	93 (16.7)	1.000	0.002
Hypertension	82 (21.9)	139 (24.9)	0.331	0.071
Prior myocardial infarction	11 (2.9)	23 (4.1)	0.445	0.064
Congestive heart failure	10 (2.7)	17 (3.0)	0.894	0.022
Chronic pulmonary disease	32 (8.6)	29 (5.2)	0.058	0.133
Charlson Comorbidity Index	0.00 (0.00, 2.00)	0.00 (0.00, 2.00)	0.077	0.167
Heart rate, bpm	97.65 (19.95)	97.11 (19.41)	0.686	0.027
Respiratory rate, bpm	28.52 (7.17)	27.39 (7.51)	0.023	0.154
CVP, mmHg	11.53 (4.81)	11.71 (5.05)	0.601	0.036
MAP, mmHg	74.89 (13.39)	80.32 (13.54)	<0.001	0.404
Vasopressors (*n*, %)	172 (46.0)	100 (17.9)	<0.001	0.631
Fluid balance, ml	2,810.77 (3,261.29)	819.08 (2,802.81)	<0.001	0.655
Hemoglobin, g/dl	9.77 (1.65)	10.00 (1.62)	0.037	0.140
Platelets, × 10^12^/L	179.63 (121.63)	200.17 (120.25)	0.013	0.170
Creatinine, mg/dl	1.35 (0.99)	1.25 (1.01)	0.151	0.098
PaO_2_/FiO_2_, mmHg	145.56 (72.57)	149.14 (60.19)	0.441	0.054
**Clinical outcomes**				
28-day mortality (*n*, %)	105 (28.1)	84 (15.1)	<0.001	0.321
VFDs by day 28, day	19.00 (14.00, 23.00)	21.00 (17.25, 24.00)	<0.001	0.315
RRT by day 90 (*n*, %)	18 (7.8)	27 (11.6)	0.215	0.130
RRT days by day 90, day	15.00 (9.00, 32.00)	13.50 (9.00, 28.50)	0.692	0.086

*Data are presented as mean (SD), median (interquartile range [IQR]), or number (proportion). APACHE III, acute physiology and chronic health evaluation III; SOFA, sequential organ failure assessment; CVP, central venous pressure; MAP, mean arterial pressure; PaO_2_/FiO_2_, ratio of partial pressure of oxygen to the fractional concentration of inspired oxygen; VFDs, ventilation free days; RRT, renal replacement therapy; SMD, standardized mean difference*.

### Association Between Diuretics Use and 28-Day Mortality

**Figure 1:**
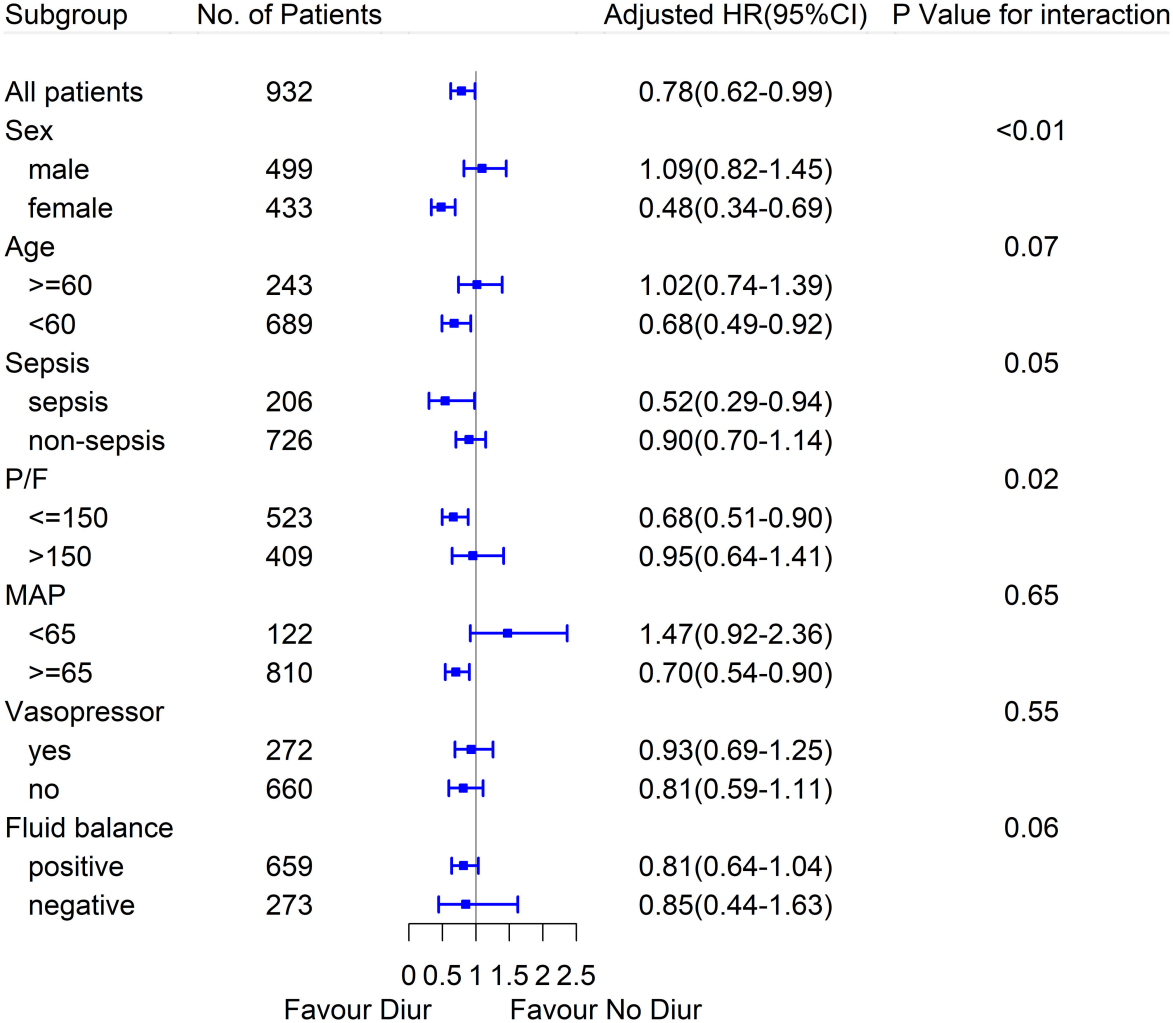
Marginal structural Cox model hazard ratio (HR) values for 28-day mortality in diuretics and no diuretics group according to the subgroups.

Time-fixed variables, such as age, APACHE III, Charlson Comorbidity Index, and the time-varying confounders (as mentioned above) were adjusted via marginal structural model. The weight distribution of IPTW applied to adjust for the confounding factors is shown in Supplementary Figure 1. Ultimately, the MSCM analysis revealed that compared with no diuretics therapy, loop diuretics use was associated with improved 28-day mortality in the patients with ARDS (HR 0.78; 95% CI 0.62–0.99; *p* = 0.04) in the overall population (Figure 1).

**Figure 2:**
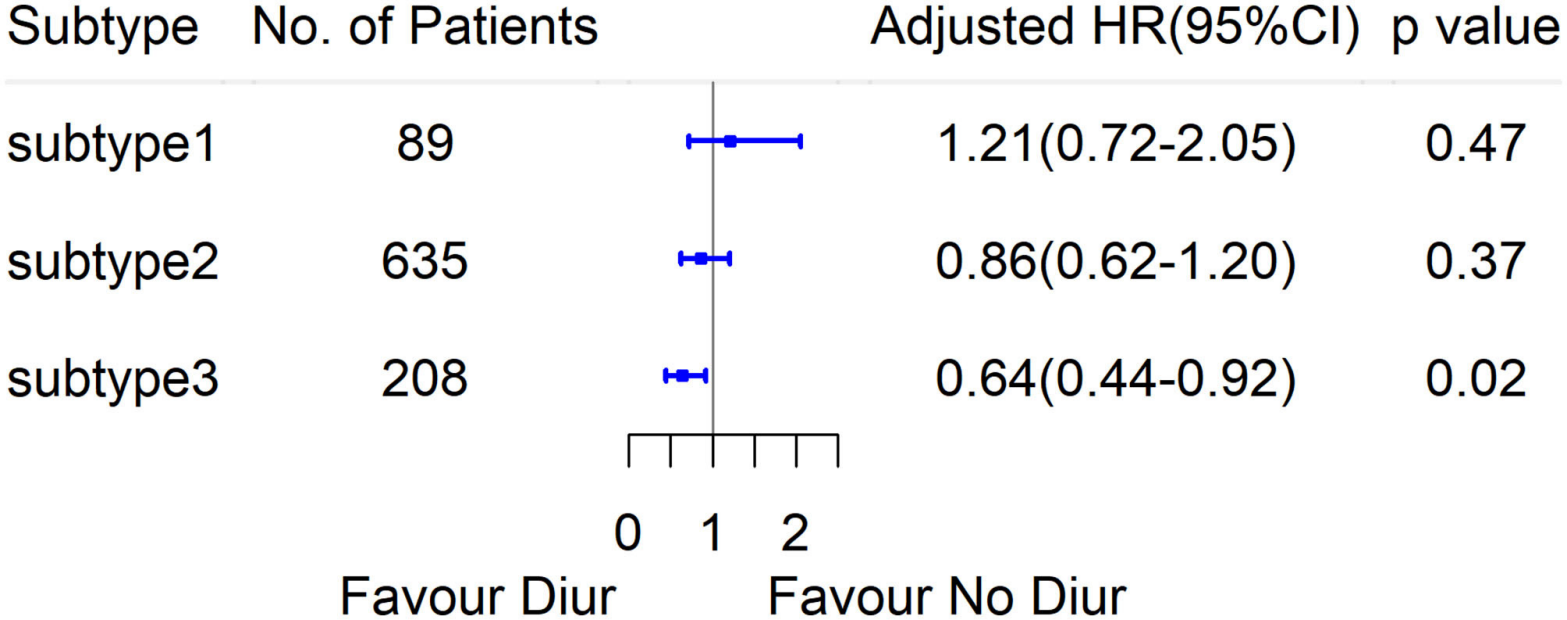
Marginal structural Cox model HR values for 28-day mortality in diuretics and no diuretics group, according to the subtypes derived by LCA.

The fit statistics of the LCA models generated are shown in Table 2. Three main phenotypes were identified, designated as subtypes 1–3. Subtype 1 included 89 (9.5%) patients that mainly suffered from pneumonia, and exhibited relatively normal renal function and the lowest CVP. Subtype 2 included 635 (68.1%) patients who were characterized by near normal serum creatinine and relatively lower CVP. Subtype 3 included 208 (22.3%) patients characterized by worse renal function, higher CVP, and higher proportions of complications, such as diabetes, hypertension, and chronic heart failure. Comparisons among the subtypes 1–3 are shown in Table 3, Supplementary Table 3, and Supplementary Figure 2. MSCM indicated that subtype three patients could be benefited from diuretics (HR 0.64; 95% CI 0.44–0.92; *p* = 0.02), whereas there were no significant associations between diuretics and 28-day mortality in subtype 1 or subtype 2 patients (Figure 2).

**Table 2:** Fit statistics for latent class analysis models.

	**BIC**	**Entropy**	***P*-values**	**Number of patients in each phenotype**			
				**1**	**2**	**3**	**4**
1	51,833			932			
2	51,009	0.746	0.10	604	328		
3	50,069	0.988	0.0001	89	635	208	
4	49,635	0.902	0.03	89	213	424	206

*BIC, Bayesian information criterion*.

**Table 3:** Comparisons of the baseline and clinical characteristics between the subtypes.

	**Subtype 1**	**Subtype 2**	**Subtype 3**	***P***
*N*	89	635	208	
Age, years	46.56 (13.22)	48.34 (16.39)	55.79 (14.23)	<0.001
Female (*n*, %)	33 (37.1)	291 (45.8)	109 (52.4)	0.045
Body mass index	24.23 (6.39)	29.19 (6.91)	30.61 (8.18)	<0.001
APACHE III	110.60 (30.04)	86.76 (28.34)	99.31 (29.53)	<0.001
Primary lung injury (*n*, %)				<0.001
Sepsis	11 (12.4)	136 (21.4)	59 (28.4)	
Trauma	1 (1.1)	68 (10.7)	4 (1.9)	
Aspiration	8 (9.0)	107 (16.9)	31 (14.9)	
Pneumonia	65 (73.0)	272 (42.8)	101 (48.6)	
Other	4 (4.5)	52 (8.2)	13 (6.2)	
**Comorbidity (** ***n*** **, %)**				
Immune suppression	14 (15.7)	30 (4.7)	27 (13.0)	<0.001
Diabetes	12 (13.5)	0 (0.0)	143 (68.8)	<0.001
Hypertension	11 (12.4)	108 (17.0)	102 (49.0)	<0.001
Myocardial infarction	1 (1.1)	12 (1.9)	21 (10.1)	<0.001
Congestive heart failure	1 (1.1)	9 (1.4)	17 (8.2)	<0.001
Chronic pulmonary disease	5 (5.6)	34 (5.4)	22 (10.6)	0.028
Charlson Comorbidity Index	6.00 (6.00, 6.00)	0.00 (0.00, 0.00)	2.00 (2.00, 3.00)	<0.001
Heart rate, bpm	99.79 (19.65)	98.13 (19.29)	93.85 (20.27)	0.011
Respiratory rate, bpm	30.20 (8.30)	27.45 (7.21)	28.03 (7.38)	0.005
CVP, mmHg	10.24 (5.91)	11.64 (4.81)	12.22 (4.85)	0.008
MAP, mmHg	74.24 (11.37)	78.95 (13.80)	77.45 (14.16)	0.008
Vasopressors (*n*, %)	0.27 (0.45)	0.27 (0.45)	0.36 (0.48)	0.071
Fluid balance, ml	2094.21 (2833.58)	1412.33 (3181.05)	2002.85 (3120.95)	0.021
Hemoglobin, g/dl	9.29 (1.55)	10.14 (1.66)	9.49 (1.44)	<0.001
Platelets, × 10^12^/L	185.94 (100.06)	192.06 (121.98)	194.40 (127.19)	0.862
Creatinine, mg/dl	1.16 (0.73)	1.21 (0.99)	1.56 (1.08)	<0.001
PaO_2_/FiO_2_, mmHg	144.68 (73.49)	147.50 (62.00)	149.69 (70.95)	0.836
**Clinical outcomes**				
28-day mortality (*n*, %)	37 (41.6)	92 (14.5)	60 (28.8)	<0.001
VFDs by day 28, day	21.00 (15.25, 24.00)	21.00 (16.00,24.00)	20.00 (16.00, 24.00)	0.922
RRT by day 90 (*n*, %)	5 (5.7)	34 (5.4)	24 (11.5)	0.008
RRT days, day	31.00 (15.00, 34.00)	16.00 (8.75, 30.00)	12.00 (9.00, 29.50)	0.391

*Data are presented as mean (SD), median (IQR), or number (proportion)*.

In the subgroup analysis, diuretics use was correlated with the reduced 28-day mortality in patients with initial MAP equal or more than 65 mmHg, and patients with PaO_2_/FiO_2_ equal or less than 150 mmHg, and no interaction was detected. Besides, the association seemed to be stronger in female patients with ARDS and sepsis-induced ARDS, and the interaction was significant. Other results of the subgroup analyses are shown in Figure 1 and Supplementary Table 4.

## Discussion

In the present study, early loop diuretics were associated with reduced 28-day mortality in patients with ARDS after adjustment for both time-fixed and time-varying confounders. LCA identified three phenotypes and patients in subtype 3 who were characterized by worse renal function and higher CVP, may benefit from diuretics. Additional subgroup analyses of the patients with ARDS indicated that associations between diuretics and reduced 28-day mortality were more marked in female patients, sepsis-induced ARDS, and patients with lower PaO_2_/FiO_2_ (≤150 mmHg), higher MAP (≥65 mmHg).

Fluid therapy is the fundamental treatment for ARDS, but volume overload is quite common and is associated with an increased risk of death (19). Diuretics are frequently prescribed to the critically ill patients to facilitate liquid removal and have become a pharmacologic adjuvant therapy in patients with ARDS (20). Studies indicate that compared with a liberal fluid strategy or standard care, conservative fluid management achieved by restricting fluid intake and the use of diuretics or hemofiltration is associated with improved oxygenation, increased ventilation-free days, and lower mortality (21–24). It has been proposed that correction of fluid retention may rely on diuretics or renal replacement therapy after the hemodynamic status is stabilized (25). Notably, early diuretics use was independently associated with lower mortality, which had been reported in a less rigorous study that used logistics regression based on the time-fixed baseline variables (7). The effects of diuretics on 28-mortality identified via the use of MSCM to adjust for time-dependent confounders further support their use in patients with ARDS.

There are evident distinctions in the etiology, physiology, and biology of patients with ARDS, leading to different responses to the same therapy (10). Three subtypes were identified by LCA in the present study, and MSCM indicated that diuretics correlated with reduced 28-day mortality in subtype 3, in which patients were characterized by elevated serum creatinine, higher CVP, and more complications, such as diabetes mellitus, hypertension, and heart disease. In a previous study, in patients with ARDS especially with concomitant acute kidney injury, positive fluid balance was associated with higher mortality (26). When used appropriately, however, frusemide may prevent and even resolve acute kidney injury as well as improving survival (27, 28). In another study, diuretics were significantly associated with lower mortality in the positive fluid balance subgroup but there was no significant association in the negative fluid balance subgroup (29). Moreover, diuretics have been recommended in patients with hypertension and heart failure to promote water and sodium excretion and reduce volume load (30, 31). The effects of diuretics on mortality might be attributed to the improvement of renal function and reduction of fluid retention.

Fluid resuscitation is highly recommended in sepsis management (32) but persistent positive fluid balance is an independent risk factor for death (33). Actually, in patients with ARDS complicated by septic shock, achieving both early goal-directed cardiovascular resuscitation and late conservative fluid therapy was reportedly associated with reduced mortality (34). A conservative fluid strategy has been recommended for sepsis-induced ARDS in which there is no evidence of tissue hypoperfusion (32). The associations between diuretics and reduced mortality in patients with sepsis-induced ARDS and those with higher MAP are consistent with the current clinical practice. Additionally, the current study indicates that diuretics may be beneficial in patients with PaO_2_/FiO_2_ ≤ 150 mmHg, possibly due to the reduction in EVLW. As EVLW estimates the fluid in pulmonary interstitial and alveolar spaces and is strongly associated with the deterioration of PaO_2_/FiO_2_, more severe lung injury, and higher mortality (3, 35), decrease in EVLW may be associated with improved survival (36). We postulated that diuretics may have substantially alleviated pulmonary edema in the worse oxygenation subgroup and contributed to better survival.

There are various pathophysiological mechanisms by which loop diuretics may improve the outcomes in patients with ARDS. Diuretics could reduce hydrostatic pressure in the event of alveolar–capillary barrier damage by limiting fluid overload, and may increase colloid osmotic pressure, resulting in reduced pulmonary edema (2, 37). Furthermore, previous studies have suggested that hyper-inflammatory or hypo-inflammatory patients respond differently to randomly assigned fluid management (9). The implementation of liberal or conservative fluid strategy may depend on the inflammatory state, whereas diuretics modify fluid balance and may thus affect the prognosis of certain subtypes.

The present study is the first to explore the effect of loop diuretics use on 28-day mortality in patients with ARDS, using MSCM to account for both time-fixed and time-dependent confounders. The phenotypes derived based on variables accessible from medical history and routine laboratory tests may inspire clinicians to implement more precise treatment. Notably, the study had several limitations. First, inflammatory biomarkers were not included in LCA due to limited access to data. Patients were divided into three categories with differences in comorbidities, CVP, and renal function, in accordance with the clinical practice. Another limitation was that we used a dataset over 15 years ago, while this still represents one of the largest randomized clinical trials investigating the effects of fluid strategy on ARDS outcomes and constitutes important evidence-based research of relevance to the guidelines and clinical practice. Last, the retrospective secondary analysis lacked the power to explain the causality. Additional well-designed randomized controlled clinical trials are required.

## Conclusion

Loop diuretics use was associated with reduced 28-day mortality in the patients with ARDS, after correction for the time-dependent variables. This association was even significant in patients with worse renal function and higher CVP, and in women, patients with sepsis-induced ARDS, and those with lower PaO_2_/FiO_2_ and higher MAP. The randomized controlled trials are required to validate these results.

## Data Availability Statement

The datasets presented in the current study are available in the BioLINCC website (https://biolincc.nhlbi.nih.gov).

## Ethics Statement

The studies involving human participants were reviewed and approved by Research Ethics Commission of Zhongda Hospital, School of Medicine, Southeast University. Written informed consent for participation was not required for this study in accordance with the national legislation and the institutional requirements.

## Author Contributions

RZ carried out the design, participated in the collection and assembly of data, and drafted the manuscript. HC wrote part of the manuscript. HC, ZG, ML, YY, and HQ participated in the manuscript revision. LL carried out the design, manuscript writing, and final approval of this research. All authors read and approved the final version before submission.

## Funding

This study was supported by the Clinical Science and Technology Specific Projects of Jiangsu Province (BE2020786 and BE2019749), the National Natural Science Foundation of China (grant number 81870066), and the Natural Science Foundation of Jiangsu Province (BK20171271).

## Conflict of Interest

The authors declare that the research was conducted in the absence of any commercial or financial relationships that could be construed as a potential conflict of interest.

## Publisher's Note

All claims expressed in this article are solely those of the authors and do not necessarily represent those of their affiliated organizations, or those of the publisher, the editors and the reviewers. Any product that may be evaluated in this article, or claim that may be made by its manufacturer, is not guaranteed or endorsed by the publisher.

## Abbreviations

HR: hazard ratio
CI: confidence of interval
APACHE III: acute physiology and chronic health evaluation III
SOFA: sequential organ failure assessment
CVP: central venous pressure
MAP: mean arterial pressure
PEEP: positive end expiration pressure
Pplat: plateau pressure
LOS: length of stay
ICU: intensive care unit
VFDs: ventilation free days
MSCM: marginal structural cox model
LCA: latent class analysis.

## References

[1] Bellani G Laffey JG Pham T Fan E Brochard L Esteban A. Epidemiology, patterns of care, and mortality for patients with acute respiratory distress syndrome in intensive care units in 50 countries. JAMA. (2016) 315:788–800. 10.1001/jama.2016.0291 26903337

[2] Neamu RF Martin GS. Fluid management in acute respiratory distress syndrome. Curr Opin Crit Care. (2013) 19:24–30. 10.1097/MCC.0b013e32835c285b 23222675

[3] Chew MS Ihrman L During J Bergenzaun L Ersson A Undén J. Extravascular lung water index improves the diagnostic accuracy of lung injury in patients with shock. Crit Care. (2012) 16:R1. 10.1186/cc10599 22214612 PMC3396226

[4] Jones SL Martensson J Glassford NJ Eastwood GM Bellomo R. Loop diuretic therapy in the critically ill: a survey. Crit Care Resusc. (2015) 17:223–6. 26282264

[5] Wiedemann HP Wheeler AP Bernard GR Thompson BT Hayden D deBoisblanc B. Comparison of two fluid-management strategies in acute lung injury. N Engl J Med. (2006) 354:2564–75. 10.1056/NEJMoa062200 16714767

[6] Cinotti R Lascarrou JB Azais MA Colin G Quenot JP Mahe PJ. Diuretics decrease fluid balance in patients on invasive mechanical ventilation: the randomized-controlled single blind, IRIHS study. Crit Care. (2021) 25:98. 10.1186/s13054-021-03509-5 33691730 PMC7943707

[7] Seitz KP Caldwell ES Hough CL. Fluid management in ARDS: an evaluation of current practice and the association between early diuretic use and hospital mortality. J Intensive Care. (2020) 8:78. 10.1186/s40560-020-00496-7 33062283 PMC7549083

[8] Bos LD Schouten LR van Vught LA Wiewel MA Ong DSY Cremer O. Identification and validation of distinct biological phenotypes in patients with acute respiratory distress syndrome by cluster analysis. Thorax. (2017) 72:876–83. 10.1136/thoraxjnl-2016-209719 28450529 PMC5964254

[9] Famous KR Delucchi K Ware LB Kangelaris KN Liu KD Thompson BT. Acute respiratory distress syndrome subphenotypes respond differently to randomized fluid management strategy. Am J Respir Crit Care Med. (2017) 195:331–8. 10.1164/rccm.201603-0645OC 27513822 PMC5328179

[10] Bos LDJ Artigas A Constantin JM Hagens LA Heijnen N Laffey JG. Precision medicine in acute respiratory distress syndrome: workshop report and recommendations for future research. EurRespir Rev. (2021) 30:200317. 10.1183/16000617.0317-2020 33536264 PMC8522998

[11] Libório AB Barbosa ML Sá VB Leite TT. Impact of loop diuretics on critically ill patients with a positive fluid balance. Anaesthesia. (2020) 75 (Suppl 1):e134–e42. 10.1111/anae.14908 31903562

[12] Wheeler AP Bernard GR Thompson BT Schoenfeld D Wiedemann HP deBoisblanc B. Pulmonary-artery versus central venous catheter to guide treatment of acute lung injury. N Engl J Med. (2006) 354:2213–24. 10.1056/NEJMoa061895 16714768

[13] Charlson ME Pompei P Ales KL MacKenzie CR. A new method of classifying prognostic comorbidity in longitudinal studies: development and validation. J Chronic Dis. (1987) 40:373–83. 10.1016/0021-9681(87)90171-8 3558716

[14] Murray JF Matthay MA Luce JM Flick MR. An expanded definition of the adult respiratory distress syndrome. Am Rev Respir Dis. (1988) 138:720–3. 10.1164/ajrccm/138.3.720 3202424

[15] Calfee CS Delucchi KL Sinha P Matthay MA Hackett J Shankar-Hari M. Acute respiratory distress syndrome subphenotypes and differential response to simvastatin: secondary analysis of a randomised controlled trial. Lancet Respir Med. (2018) 6:691–8. 10.1016/S2213-2600(18)30177-2 30078618 PMC6201750

[16] Kim SY. Determining the number of latent classes in single- and multi-phase growth mixture models. Struct Equation Model. (2014) 21:263–79. 10.1080/10705511.2014.882690 24729675 PMC3979564

[17] Xie D Yang W Jepson C Roy J Hsu JY Shou H. Statistical methods for modeling time-updated exposures in cohort studies of chronic kidney disease. Clin J Am Soc Nephrol. (2017) 12:1892–9. 10.2215/CJN.00650117 28818846 PMC5672960

[18] Naimi AI Moodie EE Auger N Kaufman JS. Constructing inverse probability weights for continuous exposures: a comparison of methods. Epidemiology. (2014) 25:292–9. 10.1097/EDE.0000000000000053 24487212

[19] van Mourik N Metske HA Hofstra JJ Binnekade JM Geerts BF Schultz MJ. Cumulative fluid balance predicts mortality and increases time on mechanical ventilation in ARDS patients: an observational cohort study. PLoS ONE. (2019) 14:e0224563. 10.1371/journal.pone.0224563 31665179 PMC6821102

[20] Munshi L Rubenfeld G Wunsch H. Adjuvants to mechanical ventilation for acute respiratory distress syndrome. Intensive Care Med. (2016) 42:775–8. 10.1007/s00134-016-4327-2 27022981

[21] Wiedermann CJ. Phases of fluid management and the roles of human albumin solution in perioperative and critically ill patients. Curr Med Res Opin. (2020) 36:1961–73. 10.1080/03007995.2020.1840970 33090028

[22] Silversides JA Major E Ferguson AJ Mann EE McAuley DF Marshall JC. Conservative fluid management or deresuscitation for patients with sepsis or acute respiratory distress syndrome following the resuscitation phase of critical illness: a systematic review and meta-analysis. Intensive Care Med. (2017) 43:155–70. 10.1007/s00134-016-4573-3 27734109

[23] Grissom CK Hirshberg EL Dickerson JB Brown SM Lanspa MJ Liu KD. Fluid management with a simplified conservative protocol for the acute respiratory distress syndrome^*^. Crit Care Med. (2015) 43:288–95. 10.1097/CCM.0000000000000715 25599463 PMC4675623

[24] Cordemans C De Laet I Van Regenmortel N Schoonheydt K Dits H Martin G. Aiming for a negative fluid balance in patients with acute lung injury and increased intra-abdominal pressure: a pilot study looking at the effects of PAL-treatment. Ann Intensive Care. (2012) 2 (Suppl 1):S15. 10.1186/2110-5820-2-S1-S15 22873416 PMC3390296

[25] Martin GS Gattinoni L Chiumello D. Fluid administration and monitoring in ARDS: which management? Intensive Care Med. (2020) 46:2252–64. 10.1007/s00134-020-06310-0 33169217 PMC7652045

[26] Zinter MS Spicer AC Liu KD Orwoll BE Alkhouli MF Brakeman PR. Positive cumulative fluid balance is associated with mortality in pediatric acute respiratory distress syndrome in the setting of acute kidney injury. Pediatr Crit Care Med. (2019) 20:323–31. 10.1097/PCC.0000000000001845 30672838 PMC6454886

[27] Joannidis M Klein SJ Ostermann M. 10 myths about frusemide. Intensive Care Med. (2019) 45:545–8. 10.1007/s00134-018-5502-4 30643933

[28] Zhao GJ Xu C Ying JC Lü WB Hong GL Li MF. Association between furosemide administration and outcomes in critically ill patients with acute kidney injury. Crit Care. (2020) 24:75. 10.1186/s13054-020-2798-6 32131879 PMC7057586

[29] Shen Y Zhang W Shen Y. Early diuretic use and mortality in critically ill patients with vasopressor support: a propensity score-matching analysis. Crit Care. (2019) 23:9. 10.1186/s13054-019-2309-9 30630521 PMC6329160

[30] Mullens W Damman K Harjola VP Mebazaa A Brunner-La Rocca HP Martens P. The use of diuretics in heart failure with congestion—a position statement from the Heart Failure Association of the European Society of Cardiology. Eur J Heart Failure. (2019) 21:137–55. 10.1002/ejhf.1369 30600580

[31] Unger T Borghi C Charchar F Khan NA Poulter NR Prabhakaran D. 2020 international society of hypertension global hypertension practice guidelines. Hypertension. (2020) 75:1334–57. 10.1161/HYPERTENSIONAHA.120.15026 32370572

[32] Rhodes A Evans LE Alhazzani W Levy MM Antonelli M Ferrer R. Surviving sepsis campaign: international guidelines for management of sepsis and septic shock: 2016. Intensive Care Med. (2017) 43:304–77. 10.1007/s00134-017-4683-6 28101605

[33] Acheampong A Vincent JL. A positive fluid balance is an independent prognostic factor in patients with sepsis. Crit Care. (2015) 19:251. 10.1186/s13054-015-0970-1 26073560 PMC4479078

[34] Murphy CV Schramm GE Doherty JA Reichley RM Gajic O Afessa B. The importance of fluid management in acute lung injury secondary to septic shock. Chest. (2009) 136:102–9. 10.1378/chest.08-2706 19318675

[35] Berkowitz DM Danai PA Eaton S Moss M Martin GS. Accurate characterization of extravascular lung water in acute respiratory distress syndrome. Crit Care Med. (2008) 36:1803–9. 10.1097/CCM.0b013e3181743eeb 18496374 PMC2713576

[36] Tagami T Nakamura T Kushimoto S Tosa R Watanabe A Kaneko T. Early-phase changes of extravascular lung water index as a prognostic indicator in acute respiratory distress syndrome patients. Ann Intensive Care. (2014) 4:27. 10.1186/s13613-014-0027-7 25593743 PMC4273855

[37] Martin GS Mangialardi RJ Wheeler AP Dupont WD Morris JA Bernard GR. Albumin and furosemide therapy in hypoproteinemic patients with acute lung injury. Crit Care Med. (2002) 30:2175–82. 10.1097/00003246-200210000-00001 12394941

