# Supplementary material for: The Effect of Loop Diuretics on 28-Day Mortality in Patients With Acute Respiratory Distress Syndrome
Source: Front Med (Lausanne). 2021 Sep 21;8:740675. doi: 10.3389/fmed.2021.740675 (PMC8490632; doi:10.3389/fmed.2021.740675)
Supplement: Supplementary file 1 [file Data_Sheet_1.doc]

Electronic supplementary material to effect of loop diuretics use on 28-day mortality of patients with ARDS

Table S1. Missing/censoring values (number, %) for included variables.

| Variables | Missing(n,%) |
| --- | --- |
| Age | 0(0) |
| Sex | 0(0) |
| Ethnic | 0(0) |
| Body mass index | 0(0) |
| APACHE III | 34(3.6) |
| SOFA | 436(46.8) |
| Charlson Comorbidity Index | 0(0) |
| Primary lung injury | 0(0) |
| Temperature | 8(0.8) |
| Heart rate | 6(0.6) |
| Respiratory rate | 13(1.4) |
| CVP | 27(2.9) |
| MAP | 8(0.8) |
| PaO2/FiO2 | 100(10.7) |
| Tidal volume | 78(8.3) |
| PEEP | 14(1.5) |
| Pplat | 133(14.2) |
| Murray lung injury score | 348(37.3) |
| Fluid balance | 15(1.6) |
| Hemoglobin | 14(1.5) |
| Platelet | 32(3.4) |
| Creatinine | 27(2.9) |
| Bilirubin | 409(43.8) |
| 28-day mortality | 0(0) |
| LOS-ICU by day28 | 198(21.2) |
| VFDs by day28 | 36(4.8) |

Abbreviations: APACHE III: Acute Physiology and Chronic Health Evaluation III; SOFA: sequential organ failure assessment; CVP: central venous pressure; MAP: mean arterial pressure; PEEP: positive end expiration pressure; Pplat: plateau pressure; LOS: length of stay; ICU: intensive care unit; VFDs: ventilation free days

Table S2. Comparisons of baseline and clinical characteristics between two groups. Data are presented as mean (SD), median (IQR), or number (proportion).

|  | No diuretics | Diuretics | *p* | SMD |
| --- | --- | --- | --- | --- |
| Catheter = PAC (n,%) | 181 (48.4) | 292 (52.3) | 0.267 | 0.079 |
| Fluid = liberal (n,%) | 266 (71.1) | 195 (34.9) | <0.001 | 0.778 |
| Ethnic (n,%) |  |  | 0.115 | 0.139 |
| White | 227 (60.7) | 375 (67.2) |  |  |
| Black | 90 (24.1) | 108 (19.4) |  |  |
| Other | 57 (15.2) | 75 (13.4) |  |  |
| Comorbidity(n, %) |  |  |  |  |
| AIDS | 37 (9.9) | 31 (5.6) | 0.018 | 0.163 |
| Solid tumor | 8 (2.1) | 7 (1.3) | 0.432 | 0.069 |
| Hepatic failure | 2 (0.5) | 6 (1.1) | 0.607 | 0.061 |
| Cirrhosis | 10 (2.7) | 22 (3.9) | 0.390 | 0.071 |
| Temperature, °C | 37.42 (0.93) | 37.50 (0.93) | 0.211 | 0.084 |
| Tidal volume, ml | 400.94 (79.50) | 394.29 (78.07) | 0.226 | 0.084 |
| PEEP, cmH_2_O | 9.00 (5.00,10.50) | 8.00 (5.00,10.00) | 0.315 | 0.085 |
| Pplat, cmH_2_O | 24.48 (7.09) | 23.85 (6.07) | 0.184 | 0.095 |
| Murray lung injury score | 2.52 (0.63) | 2.52 (0.59) | 0.905 | 0.010 |
| Bilirubin, mg/dl | 0.70 (0.40, 1.90) | 0.70 (0.50, 1.40) | 0.928 | 0.082 |
| Clinical outcomes |  |  |  |  |
| LOS- ICU by day28, day | 11.00 (6.75, 19.00) | 9.00 (6.00, 14.00) | 0.002 | 0.272 |
| Status at day 90(n, %) |  |  | <0.001 | 0.324 |
| Alive | 212 (56.7) | 398 (71.3) |  |  |
| Dead | 127 (34.0) | 114 (20.4) |  |  |
| Unknown | 35 (9.4) | 46 (8.2) |  |  |
| Failure to discharge from ICU | 104 (27.8) | 90 (16.1) | <0.001 | 0.285 |
| Failure to wean from MV | 104 (27.8) | 82 (14.7) | <0.001 | 0.325 |

PAC: pulmonary artery catheter; PEEP: positive end expiration pressure; Pplat: plateau pressure; LOS- ICU:length of ICU stay; MV: mechanical ventilation; SMD：Standardized mean differences.

Table S3. Comparisons of baseline and clinical characteristics between three subtypes. Data are presented as mean (SD), median (IQR), or number (proportion).

|  | subtype1 | Subtype2 | Subtype3 | *p* |
| --- | --- | --- | --- | --- |
| Catheter = PAC (n,%) | 38 (42.7) | 322 (50.7) | 113 (54.3) | 0.185 |
| Fluid = liberal (n,%) | 52 (58.4) | 318 (50.1) | 91 (43.8) | 0.059 |
| Ethnic (n,%) |  |  |  | <0.001 |
| White | 35 (39.3) | 443 (69.8) | 124 (59.6) |  |
| Black | 36 (40.4) | 113 (17.8) | 49 (23.6) |  |
| Other | 18 (20.2) | 79 (12.4) | 35 (16.8) |  |
| Comorbidity(n, %) |  |  |  |  |
| AIDS | 68 (76.4) | 0 (0.0) | 0 (0.0) | <0.001 |
| Solid tumor | 15 (16.9) | 0 (0.0) | 0 (0.0) | <0.001 |
| Hepatic failure | 2 (2.2) | 1 (0.2) | 5 (2.4) | 0.003 |
| Cirrhosis | 7 (7.9) | 0 (0.0) | 25 (12.0) | <0.001 |
| Temperature, °C | 36.94 (0.94) | 37.56 (0.89) | 37.42 (0.99) | <0.001 |
| Tidal volume, ml | 397.10 (79.20) | 399.32 (80.28) | 389.56 (73.13) | 0.335 |
| PEEP, cmH2O | 10.00 (8.00,12.00) | 8.00 (5.00,10.00) | 8.00 (5.00,10.00) | 0.295 |
| Pplat, cmH2O | 24.62 (5.80) | 24.12 (6.54) | 23.83 (6.70) | 0.666 |
| Murray lung injury score | 2.56 (0.61) | 2.52 (0.61) | 2.51 (0.62) | 0.844 |
| Clinical outcomes |  |  |  |  |
| LOS- ICU by day28, day | 9.00 (4.75,16.00) | 9.00 (6.00,15.00) | 11.00 (7.00,17.00) | 0.105 |
| Status at day 90(n, %) |  |  |  | <0.001 |
| Alive | 37 (41.6) | 465 (73.2) | 108 (51.9) |  |
| Dead | 43 (48.3) | 119 (18.7) | 79 (38.0) |  |
| Unknown | 9 (10.1) | 51 (8.0) | 21 (10.1) |  |
| Failure to discharge from ICU | 33 (37.1) | 95 (15.0) | 66 (31.7) | <0.001 |
| Failure to wean from MV | 35 (39.3) | 91 (14.3) | 60 (28.8) | <0.001 |

Table S4. MSCM and subgroup analyses of 28-day mortality between diuretics and no diuretics group

| Variable | No.of patients | Adjusted HR (95%CI) | HR | *p* for interaction |
| --- | --- | --- | --- | --- |
| All patients | 932 | 0.78(0.62-0.99) | 0.78 |  |
| Sex |  |  |  | <0.01 |
| male | 499 | 1.09(0.82-1.45) | 1.09 |  |
| female | 433 | 0.48(0.34-0.69) | 0.48 |  |
| Age |  |  |  | 0.07 |
| >=60 | 243 | 1.02(0.74-1.39) | 1.02 |  |
| <60 | 689 | 0.68(0.49-0.92) | 0.68 |  |
| Sepsis |  |  |  | 0.05 |
| sepsis | 206 | 0.52(0.29-0.94) | 0.52 |  |
| non-sepsis | 726 | 0.90(0.70-1.14) | 0.90 |  |
| P/F |  |  |  | 0.02 |
| <=150 | 523 | 0.68(0.51-0.90) | 0.68 |  |
| >150 | 409 | 0.95(0.64-1.41) | 0.95 |  |
| MAP |  |  |  | 0.65 |
| <65 | 122 | 1.47(0.92-2.36) | 1.47 |  |
| >=65 | 810 | 0.70(0.54-0.90) | 0.70 |  |
| Vasopressor |  |  |  | 0.55 |
| yes | 272 | 0.93(0.69-1.25) | 0.93 |  |
| no | 660 | 0.81(0.59-1.11) | 0.81 |  |
| Fluid balance |  |  |  | 0.06 |
| positive | 659 | 0.81(0.64-1.04) | 0.81 |  |
| negative | 273 | 0.85(0.44-1.63) | 0.85 |  |
| Subtype |  |  |  |  |
| subtype1 | 89 | 1.21(0.72-2.05) | 1.21 |  |
| subtype2 | 635 | 0.86(0.62-1.20) | 0.86 | 0.31 |
| subtype3 | 208 | 0.64(0.44-0.92) | 0.64 | 0.12 |

Abbreviations: HR: hazard ratio, MAP: mean arterial pressure, P/F:PaO_2_/FiO_2_.





Figure S1. Weights distribution plot for the inverse probability weights that were used to adjust for confounding-Log distribution.


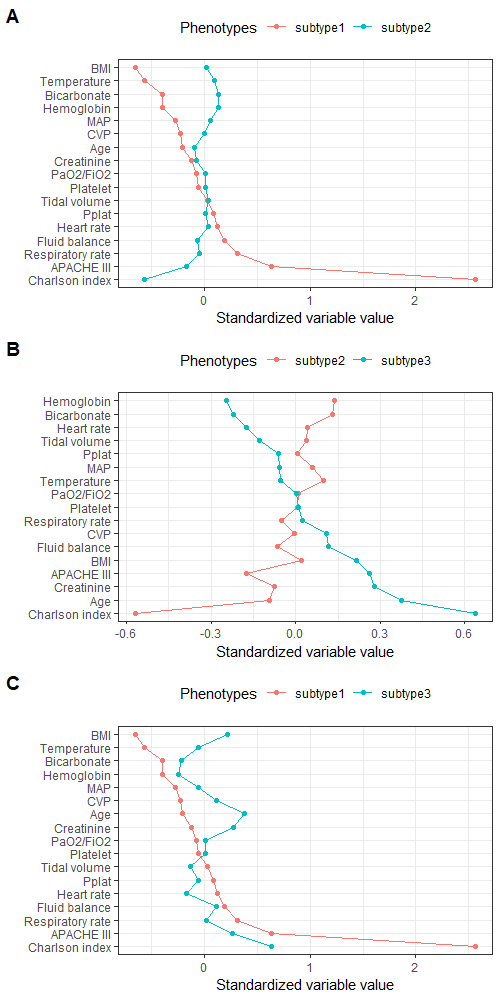


Figure S2.Differences in the standardized values of each variable by phenotype on the x-axis, with the individual continuous variables along the y-axis.
